# A new A-P compartment boundary and organizer in holometabolous insect wings

**DOI:** 10.1038/s41598-017-16553-5

**Published:** 2017-11-27

**Authors:** Roohollah Abbasi, Jeffrey M. Marcus

**Affiliations:** Department of Biological Sciences, University of Manitoba, Winnipeg, MB Canada

**Keywords:** Ectoderm, Evolutionary developmental biology, Development, Entomology

## Abstract

Decades of research on the highly modified wings of *Drosophila melanogaster* has suggested that insect wings are divided into two Anterior-Posterior (A-P) compartments separated by an axis of symmetry. This axis of symmetry is created by a developmental organizer that establishes symmetrical patterns of gene expression that in turn pattern the A-P axis of the wing. Butterflies possess more typical insect wings and butterfly wing colour patterns provide many landmarks for studies of wing structure and development. Using eyespot colour pattern variation in *Vanessa* butterflies, here we show an additional A-P axis of symmetry running between wing sectors 3 and 4. Boundaries of *Drosophila* mitotic clones suggest the existence of a previously undetected Far-Posterior (F-P) compartment boundary that coincides with this additional A-P axis. A similar compartment boundary is evident in butterfly mosaic gynandromorphs. We suggest that this additional compartment boundary and its associated developmental organizer create an axis of wing colour pattern symmetry and a gene expression-based combinatorial code, permitting each insect wing compartment to acquire a unique identity and allowing for the individuation of butterfly eyespots.

## Introduction

The anterior-posterior (A-P) axis of *Drosophila melanogaster* wings is typically divided into two developmental wing compartments that were first discovered by genetic mosaic analysis using mitotic clones^1^. The posterior compartment was later shown to coincide with a domain of expression of the transcription factor engrailed^2^. It has been widely believed that the A-P axis of all insect wings is organized in the same way, even though *Drosophila* wings are highly modified and reduced in size and in wing vein complexity compared to most other insect species (Extended Data Fig. 1)^3^.

**Figure 1:**
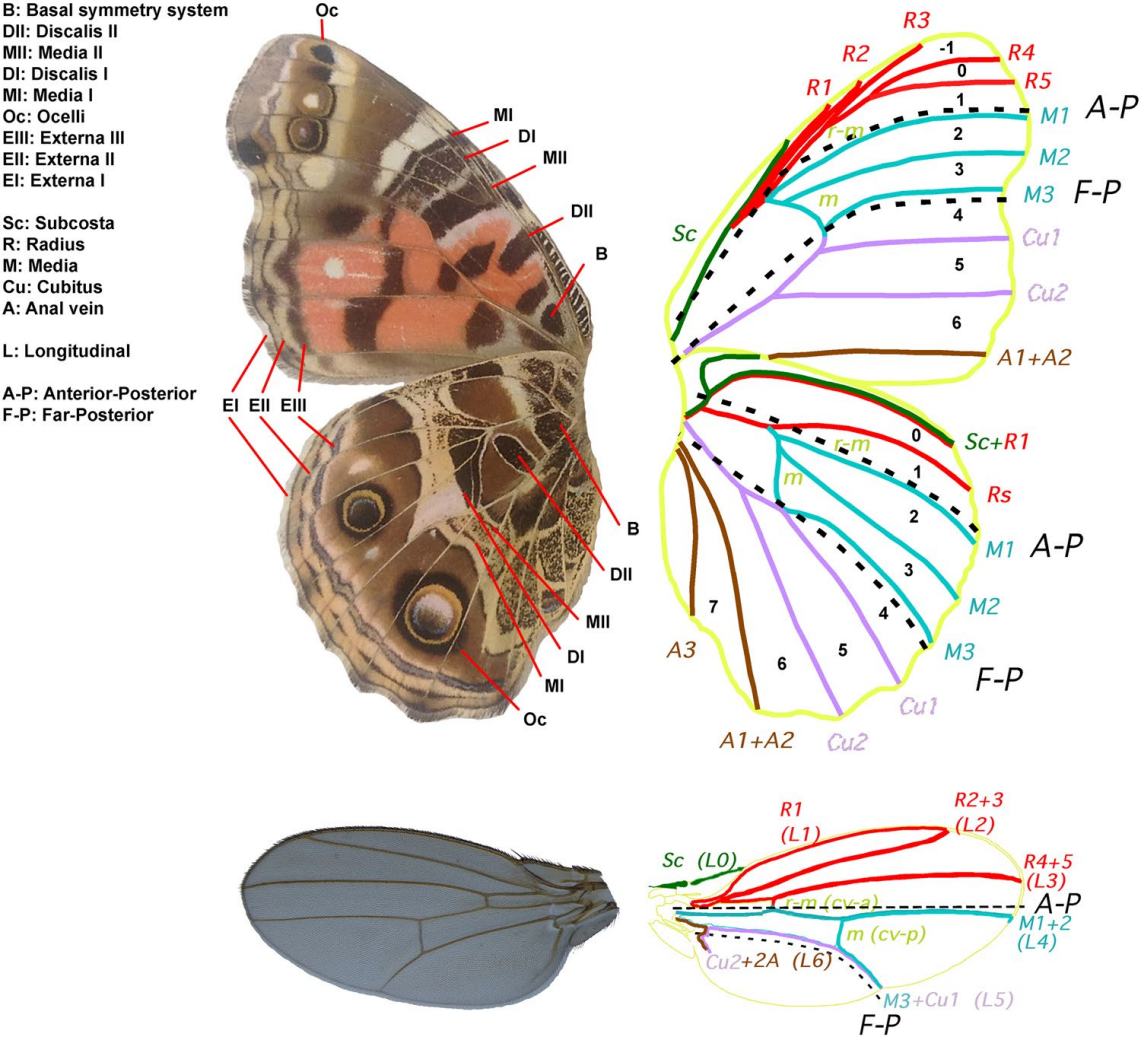
The Nymphalid groundplan and vein homologies between butterflies and *Drosophila*. Major butterfly colour pattern elements (like in *Vanessa braziliensis*, ventral surfaces) are: Basalis (B); Discalis II (DII); Media II (MII); Discalis I (DI); Media I (MI); Border ocelli (Oc); and Externa (E): including parafocal (EIII), submarginal (EII), and marginal (EI) elements^9,90^. The wing veins of *Vanessa* (Subcosta (Sc); Radius (R); Media (M); Cubitus (Cu); and Anal vein (A)^9,91^) are compared with veins in *Drosophila melanogaster* (labelled with both conventional *Drosophila* vein names (L1-L5) and butterfly homologues^92^). The A-P and F-P compartment boundaries are indicated by dotted lines.

The morphology of butterfly wings is far more representative of other holometabolous insect wings and also feature numerous colour pattern landmarks (Fig. 1)^4^. Eyespot and non-eyespot colour patterns in butterflies are evolutionarily recent serially homologous features^4^ that are important models for understanding the evolution and development of morphological structures^5–7^. Eyespots and other insect colour patterns are specified during development in the context of pre-existing developmental-genetic architecture responsible for defining the placement of wing margins and wing veins^8^. Colour pattern location and organization often reflects positional information provided by this architecture^9^. The series of eyespots found on the wings of many butterflies are perhaps the best studied of all insect colour patterns^10^. While serially homologous, the eyespots are also individuated, meaning that they can differ substantially from one another even between adjacent eyespots on the same wing surface^6^. Much of what is known about the genetic mechanism for individuation of eyespots comes from epistatic interactions between butterfly *Bicyclus anynana* mutants to produce specific eyespot phenotypes. *Bicyclus anynana* shows a developmental-genetic association between eyespots 3 and 4, which appear and disappear in parallel in different mutant backgrounds on both the dorsal forewing and the ventral hindwing^11–13^. Using a phylogenetic approach to make colour pattern comparisons between species of *Junonia* butterflies, Kodandaramaiah^14^ found a strong association between the presence of eyespots 2 and 5 on the dorsal hindwing. Physiological perturbations during the development of *Vanessa cardui* butterfly wings reveals phenotypic associations between eyespots 3 and 4 as well as between eyespots 2 and 5^15^. The generality of any of these eyespot associations has been unknown, as have the mechanisms that may be producing these associations.

Here we re-examined these eyespot associations by asking whether genetically associated eyespots also individuate in similar ways. We addressed this question by examining the eyespot morphology of 22 *Vanessa* butterfly species, a genus known for highly diverse eyespot phenotypes^6^, and determining whether these proposed morphological associations could be generalized for all wing surfaces. We took a phylogenetic approach to the study of eyespot colour patterns^6,14^ and conducted a statistical analysis using independent contrasts^16^ to detect eyespot morphology correlations in *Vanessa* butterflies. In *Drosophila* wings, the A-P wing compartment boundary has an associated developmental organizer that produces symmetrical gene expression domains on either side of the boundary^17^. We hypothesized the existence of an additional compartment boundary and a developmental organizer in the posterior portion of the wing that may be responsible for the symmetrical eyespot associations found in *Vanessa* in this study and in other species by previous authors^12–15^. We then used the fruit fly *Drosophila melanogaster* as an experimental model and examined naturally occurring lepidopteran homeotic mosaics and mosaic gynandromorphs to test for the existence of this additional compartment boundary. Finally, we propose a developmental model for eyespot individuation and insect wing A-P patterning.

## Results and Discussion

### Independent contrast analysis of ocelli

Independent contrast (IC) analysis is a phylogenetic method that calculates correlations between traits in related taxa, by transforming the data so as to remove similarity caused by phylogenetic relatedness^16^. IC was used to evaluate correlations between characters on the same and different wing surfaces (dorsal, ventral) and between characters on the same and different wings (forewing, hindwing). Comparing the number of components in homologous eyespots between the dorsal and ventral sides of the forewing and hindwing revealed only one significant positive correlation each (forewing: r = 0.57, p = 0.0045, hindwing r = 0.46, p = 0.0272) between the eyespots in wing sector 5. Comparing the number of eyespots between the two sides of the forewing revealed that they are significantly correlated (r = 0.46, p = 0.0272), but the same comparison on the hindwing did not reveal any significant correlations.

Of the 28 comparisons investigated within each wing surface, many significant correlations were detected, but these were generally limited to interactions on a single wing surface (Table 1). Most of the single-wing-surface correlations were between eyespots in adjacent wing sectors, suggesting that these relationships may be relatively evolutionarily plastic. The only significant positive correlations found on every wing surface were between eyespots 2 and 5 (2 + 5 correlation) and between eyespots 3 and 4 (3 + 4 correlation) (Table 1).

**Table 1:** Significant independent contrast correlations detected among the 28 within-wing-surface comparisons.

	Dorsal surface	Ventral surface
Forewing	−1 + 3 (r = 0.81, p = 2.82 × 10^−6^)	−1 + 0 (r = 0.95, p = 4.37 × 10^−12^)
	−1 + 4 (r = 0.57, p = 0.0045)	−1 + 1 (r = 0.53, p = 0.0093)
	0 + 3 (r = 0.81, p = 2.82 × 10^−6^)	−1 + 2 (r = 0.53, p = 0.0093)
	0 + 4 (r = 0.57, p = 0.0045)	0 + 1 (r = 0.48, p = 0.0204)
	1 + 4 (r = 0.58, p = 0.0037)	0 + 2 (r = 0.48, p = 0.0204)
	1 + 5 (r = 0.47, p = 0.0236)	1 + 5 (r = 0.48, p = 0.0204)
	2 + 4 (r = 0.58, p = 0.0037)	**2 + 5 (r = 0.48, p = 0.0204)**
	**2 + 5 (r = 0.47, p = 0.0236)**	**3 + 4 (r = 0.51, p = 0.0129)**
	**3 + 4 (r = 0.49, p = 0.0176)**	3 + 6 (r = 0.46, p = 0.0272)
		4 + 5 (r = 0.53, p = 0.0093)
		4 + 6 (r = 0.51, p = 0.0129)
		5 + 6 (r = 0.54, p = 0.0078)
Hindwing	**2 + 5 (r = 0.48, p = 0.0204)**	1 + 3 (r = 0.66, p = 0.0006)
	**3 + 4 (r = 0.69, p = 0.0003)**	1 + 4 (r = 0.67, p = 0.0005)
	4 + 5 (r = 0.79, p = 7.373 × 10^−6^)	1 + 5 (r = 0.52, p = 0.0110)
		**2 + 5 (r = 0.53, p = 0.0093)**
		**3 + 4 (r = 0.82, p = 1.675 × 10** ^**−6**^ **)**
		3 + 5 (r = 0.44, p = 0.0356)
		4 + 7 (r = 0.54, p = 0.0078)
		6 + 7 (r = 0.48, p = 0.0204)

Significant positive correlations found on all wings surfaces are indicated in bold.

Butterfly eyespots have roles in mate selection and predator avoidance, with different wing surfaces often being specialized for different purposes^6,18,19^. Consequently, selective forces often drive colour pattern phenotypes present on different wing surfaces in different directions, so that each wing surface is phenotypically distinct.

Eyespots 2, 3, 4, and 5 are expected to be subject to these selective forces like all other colour patterns, yet the significant positive phenotypic correlations among eyespots 2, 3, 4, and 5 are very consistent in *Vanessa*. These observations, in combination with recent work examining physiological perturbations in wild-type *Vanessa cardui* ^15^ and with prior observations of similar eyespot correlation patterns in *Junonia* and *Bicyclus* ^12–14^, suggests that these patterns of eyespot correlations may reflect the underlying developmental architecture of the insect wing. This axis of colour pattern symmetry is suggestive of an A-P colour pattern organizer in the vicinity of vein M3 in the far posterior of the wing, which runs between wing sectors 3 and 4 (Fig. 1) and which does not coincide with the position of the A-P organizer and compartment boundary known from *Drosophila* (Fig. 1).

### Clonal analysis of *Drosophila* wings

We used *Drosophila melanogaster* to test the hypothesis of the existence of a second compartment boundary in the far posterior of the insect wing, by generating and mapping mitotic clones (Extended Data Fig. 2)^20^. In *Drosophila* mosaics, clones do not cross the A-P wing compartment boundary defined by *engrailed* expression^21^, so experimentally marked clones would not be expected to cross a compartment boundary in the far posterior portion of the wing either. We designed two different genetic crosses to produce mitotic clones with visible wing cuticle markers (Extended Data Fig. 3).

**Figure 2:**
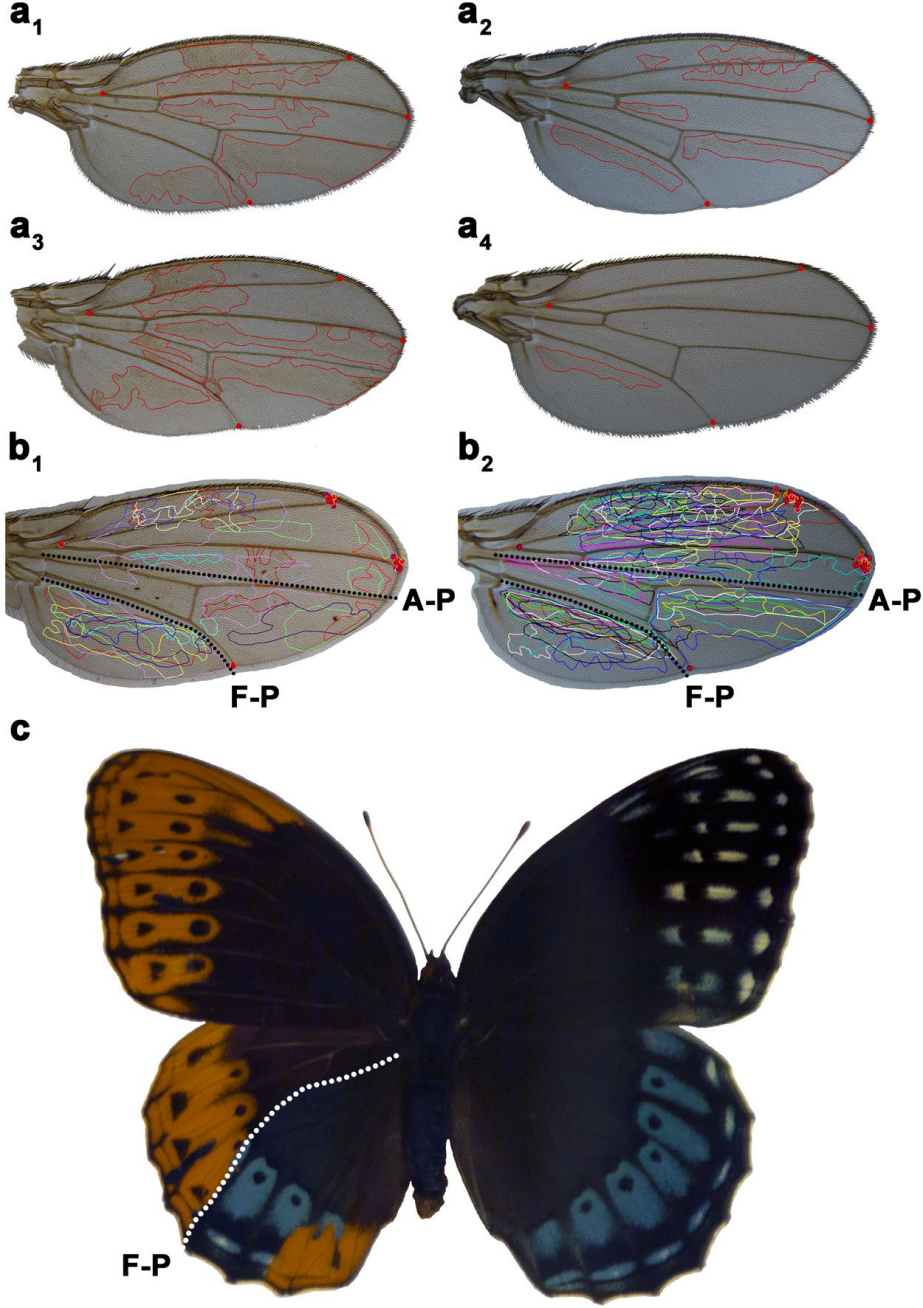
Mosaic analysis. Representative *Drosophila* wings with mutant *yellow* (y) clones produced by the FLP/FRT system (**a**_**1**_ **–a**_**4**_). Clones do not cross and have smooth edges along compartment boundaries. Superimposing all wings with mitotic clones produced by FLP/FRT on chromosome 1 (**b**_**1**_) or on chromosome 2 (**b**_**2**_) (red dots indicate landmarks used to align wings) show consistent positions of the A-P and F-P compartment boundaries in *Drosophila* wings. Large clones in mosaic gynandromorph butterflies such as this *Speyeria diana* ^24^ (**c**) also reveal a well-defined F-P boundary. Male wing scales are orange and female scales are blue.

Wings from a total of 889 female flies were collected from these crosses and mounted on microscope slides. Of 1778 wings examined, 44 wings had large mitotic clones (12/554 wings from chromosome 1 clones, and 32/896 wings from chromosome 2 clones, Extended Data Fig. 3). The wings with large clones from each cross (Fig. 2a) were photographed and superimposed on top of each other, optimizing fit for the 2 posterior-most landmarks (Fig. 2b). Mitotic wing clones never cross an apparent Far-Posterior (F-P) compartment boundary just posterior of *Drosophila* vein L5, which is equivalent and homologous to vein M3 in butterfly wings (see Fig. 1. for vein homologies). The position of this compartment boundary coincides with and is homologous to the location of the axis of symmetry between wing sectors 3 and 4 identified from *Vanessa* eyespot correlations. In this experiment, mitotic clones also did not cross the A-P boundary as shown in the images of individual wings (Fig. 2a). However, the process we used to superimpose all of the wing images optimizing fit in the posterior of the wing to clearly illustrate the F-P boundary also introduces some geometric distortion in the anterior of the wing images and partially obscures the well-studied A-P boundary that is also present (Fig. 2b).

### Lepidopteran mosaic gynandromorphs and homeotic mosaics

Mitotic clones are difficult to generate experimentally in the Lepidoptera, but rare naturally occurring mosaic gynandromorphs^22^ and homeotic mosaics^23^ are well known in the Lepidoptera. A small subset of these mosaics feature clones large enough and appropriately located on the wing to be informative with respect to the Far-Posterior compartment boundary hypothesis. Specimens with large clones are often visually striking (Fig. 2c) and many have been described and illustrated in the literature^22–24^. Reviewing a large number of published specimens with appropriately placed large mitotic clones shows that these large-cloned cellular mosaics consistently respect a compartment boundary in the same location as identified from *Vanessa* and *Drosophila*.

Collectively, these findings suggest that there is a previously unrecognized developmental compartment boundary in the posterior portion of the wing in holometabolous insects, the Far-Posterior (F-P) compartment boundary (Figs 1, 2). Given that the A-P compartment boundary was discovered approximately four decades ago^20^ and that *Drosophila* is a well-studied model, our finding of an additional, previously undocumented compartment boundary can be considered a breakthrough in the understanding of insect wing development and patterning.

### Combinatorial model for the wing A-P axis

**Figure 3:**
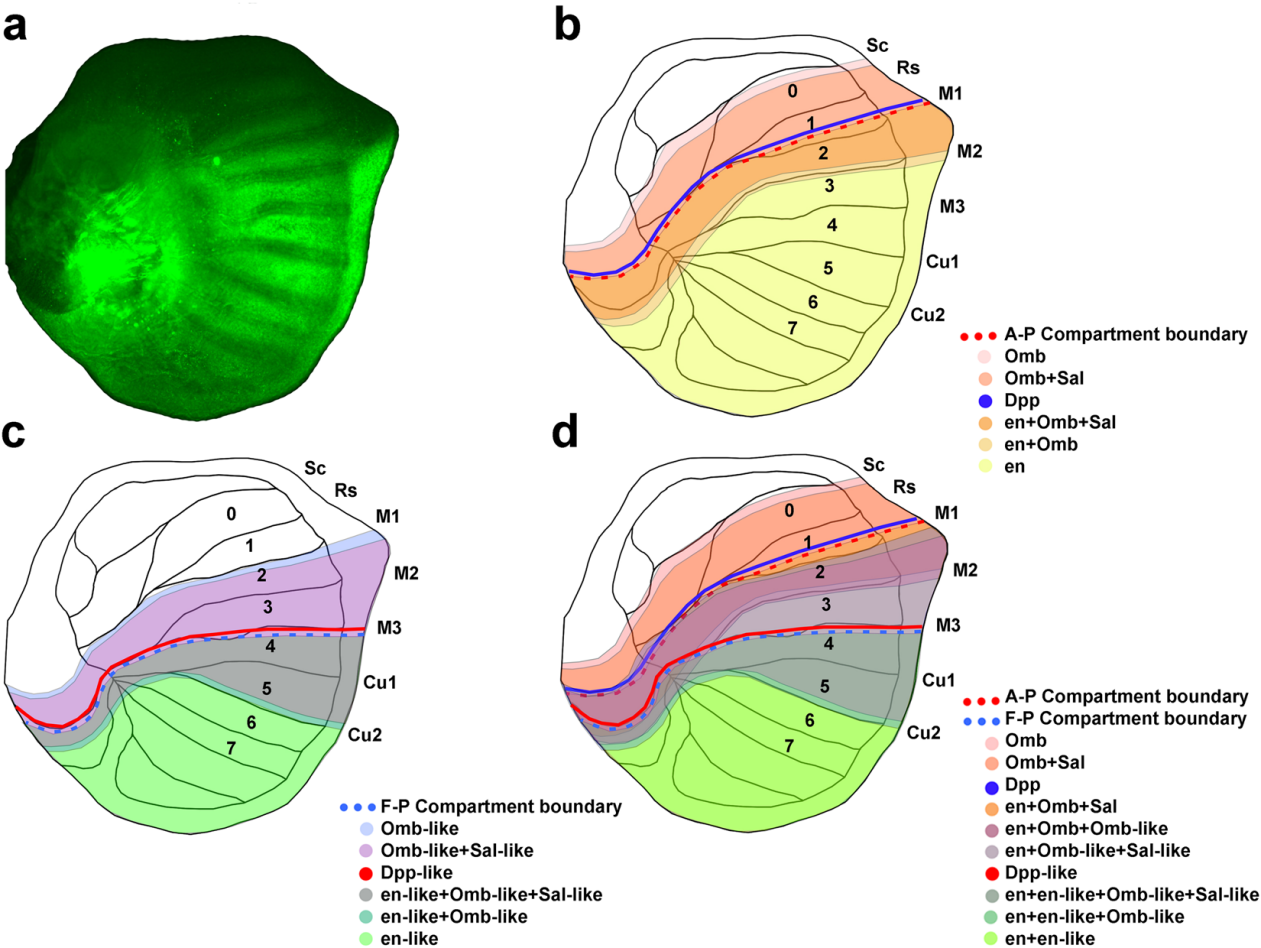
Proposed model for the compartment boundaries on butterfly wings. (**a)** Expression pattern of engrailed protein on a *Junonia* wing disc. (**b)** Model of the well-studied A-P compartment boundary associated with *engrailed* expression^25,33–35^. (**c)** The additional hypothetical F-P compartment boundary proposed based on our independent contrast analysis of eyespot phenotypes. (**d)** Combining the patterns of gene expression from the two compartment boundaries. Each wing sector is characterized by a unique combination of gene expression, providing a potential mechanism by which the eyespots found in each wing sector can be independently regulated.

Butterfly eyespot development occurs in the late larval and pupal stages and is superimposed on a pre-existing molecular genetic coordinate system that is responsible for regulating the development of the wing as a whole^25,26^. Much of what is known about this coordinate system has come from research on *Drosophila*, which has established that the A-P patterning of the wing is organized by a domain of expression of the *engrailed (en)* transcription factor in cells in the posterior portion of the wing^8,27^. Cells in this region also secrete the short-range signal *hedgehog* (*hh*), which in turn stimulates the expression of the long-range signal *decapentaplegic* (*dpp*) in cells immediately anterior to the *engrailed* expression domain^28^. The concentration of *dpp* received by cells in the wing establishes a set of nested domains of gene expression, such as *spalt* (*sal*) and *optomotor-blind* (*omb*), which define the placement of wing veins in *Drosophila* ^17,27,29^. Veins are essential for the proper development of butterfly colour patterns and it appears that the developmental processes responsible for placing eyespots and veins on wings are inter-related^30–32^. Wing veins also define the boundaries of entomological wing sectors (fields of cytological cells bordered by the wing margin and a series of wing veins)^31^. The existence of the *en* A-P compartment boundary in butterfly wings has been documented by examination of *en* and *sal* expression patterns^25,33–35^. Our experiment showing *en* expression in the imaginal wing disc of 5th instar *Junonia coenia* larvae places the A-P compartment boundary in the region between veins M1 and M2 on the wing disc, with engrailed expressed posterior to the compartment boundary (Fig. 3a). Expression of *dpp* and *omb* has also been documented in transcriptome analysis of the wing disc of the moth *Ostrinia furnacalis* ^36^, but there are no available data pertaining to the expression domains of *dpp* and *omb* in butterflies and moths. Based on known expression domains of *en* and *sal* in butterflies, it is possible to project the expected domains of gene expression for the remaining genes responsible for A-P patterning in *Drosophila* onto a butterfly wing (Fig. 3b).

If we continue to use the *en* compartment boundary and *dpp* organizer in the anterior of the *Drosophila* wing as a model for the Far-Posterior compartment boundary, this suggests that the placement of the posterior organizer in the butterfly wing may also be specified by a compartment boundary associated with the anterior limit of expression of a gene with a role similar to *en* (*en-like)*, thereby defining a far-posterior compartment^28^. Given the vast amount of experimental work that has been devoted to the development of the *Drosophila* wing, it is important to note that there are no published precedents for the existence of an additional wing compartment in that model system^17^. However, the wings of *Drosophila*, like those of all flies, are highly derived in structure with vestigial hindwings (converted to halteres) and forewings that have reduced venation and are compressed along the A-P axis, with most of the apparent tissue loss from the posterior portions of the wing (Extended Data Fig. 1). This hypothetical posterior boundary and organizer may drive gene expression in the posterior portion of the wing, producing a nested series of domains of gene expression organized symmetrically around it, similar to the way in which known patterns of gene expression are arranged around the *dpp* organizer in the anterior portion of the wing (Fig. 3c). This would provide a mechanistic explanation for the consistent tendency of Nymphalid butterflies to produce eyespots in a mirror image arrangement (with parallel phenotypes for eyespots 3 + 4 and 2 + 5) around the wing vein that separates wing sectors 3 and 4. A ligand with properties similar to *dpp* (*dpp-like*) may serve as the organizing signal and genes with functional similarities to *sal*, *omb*, and *brinker* (*brk*) (*sal-like*, *omb-like*, *brk-like*) may establish the nested domains of gene expression. Whether any of these hypothetical genes have sequence homology to those that participate in the *en* compartment boundary and the *dpp-*dependent A-P wing organizer is a matter of speculation. In A-P body axis determination in the *Drosophila* embryo, many genes (primarily the *Hox* genes) responsible for regional specification of body segments share sequence homology, but other genes with very similar roles (e.g. *teashirt* (*tsh*)) have no sequence homology to the *Hox* genes^37^.

Many of the other insect species in which wing development has been studied, such as ants (Hymenoptera)^38^ and beetles (Coleoptera)^39^ also happen to have wings that are highly modified in structure and/or are reduced in size (modified and reduced venation in ants, transformation of the forewings into hardened elytra in beetles). While lepidopteran wings are unique among insects in that they are covered with scales, the wings of butterflies and moths are otherwise more structurally representative of typical insect wings than other species whose wing development has been studied to date (Extended Data Fig. 1)^40^. It is possible that the existence of a far-posterior compartment and its associated organizer is a novel developmental trait in the Holometabolous insect lineage that gave rise to the Diptera and the Lepidoptera. In this scenario, the Lepidoptera may have evolved to have an enlarged Far-Posterior developmental compartment and enlarged posterior portions of the wings for improved flight performance, visual signalling, thermoregulation, or pheromone secretion, all functions of the wings of extant species of Lepidoptera^18,19^. Alternatively, it is possible that the Far-Posterior wing compartment has not been detected in some other insects because it has been lost over evolutionary time as their wings have been modified and reduced in size. Finally, it is also possible that the Far-Posterior wing compartment is so reduced in these species that mutations that affect this compartment do not have large phenotypic effects in model systems like *Drosophila*, making mutants difficult to identify in mutagenesis screens^41^. Of these alternatives, we believe this last hypothesis is the most likely to be correct because of the consistent organization of homologous wing veins that appear to be associated with the Far-Posterior compartment across all insect orders, and the small size of this compartment in *Drosophila*, by far the most extensively studied model species for insect wing development (Extended Data Fig. 1).

**Figure 4:**
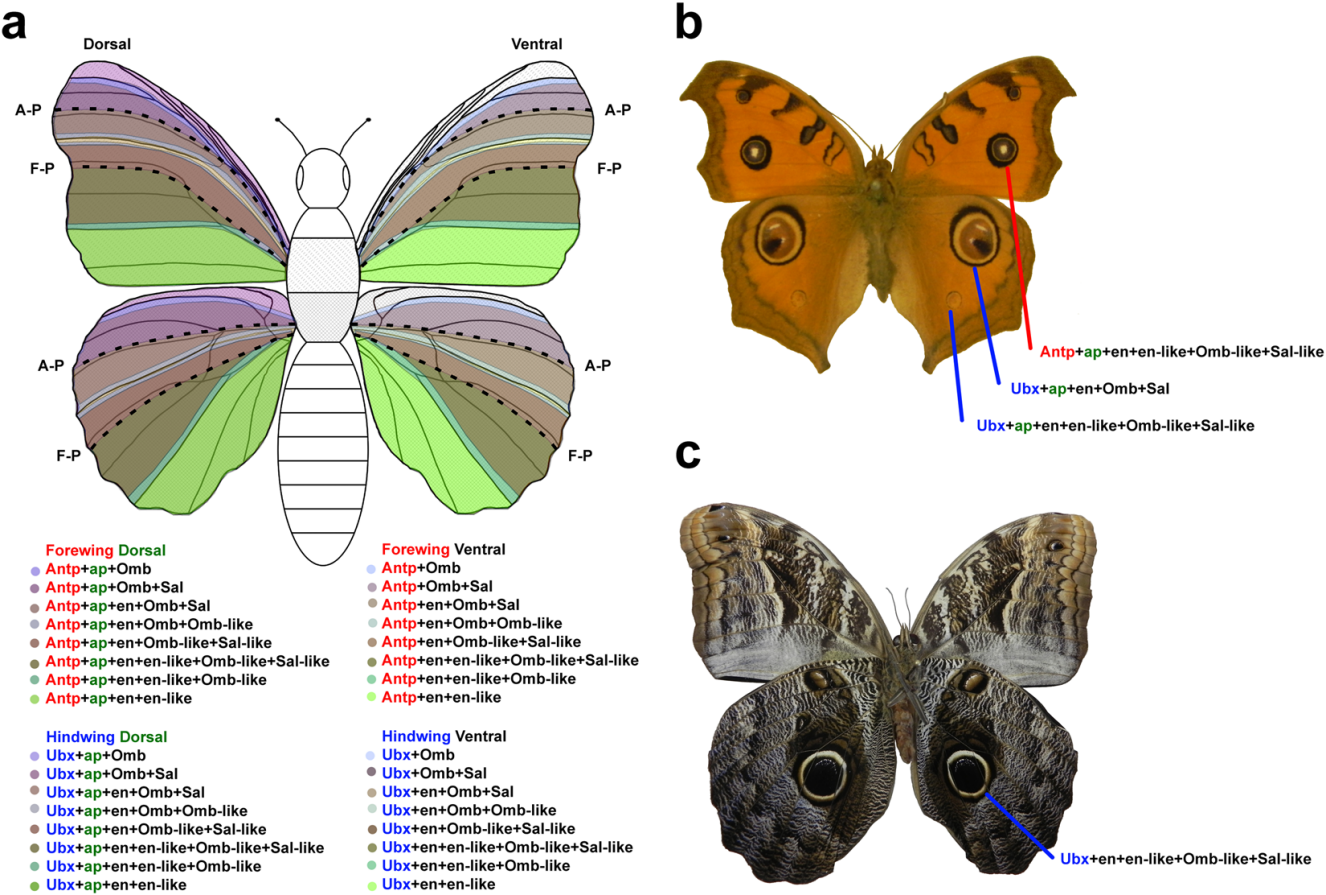
Combinatorial genetic system for individuating each wing compartments. (**a**) Each wing compartment on both the dorsal and ventral surfaces of the forewings and hindwings has a unique combination of expressed genes, allowing for the independent regulation of colour pattern phenotypes such as eyespots in each sector. (**b**) Dorsal wing surfaces of *Junonia almana*, with the combination of expressed genes predicted to be associated with 3 prominent eyespots. (**c**) Ventral wing surfaces of *Caligo placidianus* with combination of expressed genes predicted to be associated with the most prominent eyespot.

A consequence of the A-P patterning mechanism that we have proposed is that the overlapping patterns of gene expression, driven by the *dpp* organizer and the newly hypothesized posterior F-P organizer, create distinct combinations of gene expression in each butterfly wing sector (Fig. 3d). We hypothesize that when combined with the transcription factor loci *apterous* (*ap*), which defines dorsal cell fates in the wing^42^, and *Ultrabithorax* (*Ubx*), which distinguishes the hindwing from the forewing^23^, the A-P patterning genes would create a unique combinatorial code or “address” for each wing sector on all 4 wing surfaces. This would allow butterflies to independently determine the phenotype of each wing sector through the regulation of downstream genes responsible for initiating colour pattern formation^31,35^. In the case of eyespots, which show enormous diversity with respect to the number and degree of elaboration on any given wing surface^7,43^, this suggests a potential mechanism by which eyespots in any wing sector can become individuated (Fig. 4a).

This hypothesis and model have some similarities to a previously proposed cis-regulatory element evolution hypothesis for eyespot diversification^12,13,44^. We suspect that much of the gene regulation within each wing sector is mediated through the differential binding of transcription factors to regulatory elements associated with genes involved in eyespot formation. There is, however, no direct evidence that all of the cis-regulatory elements responsible for eyespot individuation are associated with a single master control gene and are organized in a manner similar to the regulatory elements of *even-skipped* (*eve*) in *Drosophila* as suggested in the original model^12,13,44^. Rather, it seems likely that the eyespot regulatory elements may be associated with several different genes at the beginning of the eyespot genetic regulatory network such as *Distal-less* (*Dll*), *Notch* (*N*), and perhaps *Antennapedia* (*Antp*)^31,45^. This would permit independent control of the initiation of eyespot development in multiple wing sectors while also retaining the ability to produce different eyespot phenotypes in each wing sector. This is done by modulating multiple interactions between genes responding to the wing A-P organizers and genes responsible for regulating eyespot development.

Remarkably, if our model is correct, it not only offers a potential explanation for why certain eyespots (e.g. 3 + 4, 2 + 5) often have similar phenotypes on a given wing surface, but also a mechanism for how these phenotypic correlations can become dissociated to produce individuated eyespots as is seen, for example, on the ventral forewing of *V*. *braziliensis* (Fig. 1), the dorsal wing surfaces of *Junonia almana* (Fig. 4b), or the ventral wing surfaces of *Caligo placidianus* (Fig. 4c). All that may be required to change the phenotypic associations between eyespots on a wing surface is alteration of the binding sites for the gene products responsible for A-P patterning of the wing in the regulatory regions of genes in the eyespot development pathway. A detailed understanding of the mechanism by which eyespots are deployed in wing sectors greatly enhances the value of this system as a model for studying the evolution of serial homology^7,43^.

## Conclusions

We studied compartment boundaries in holometabolous insects using *Vanessa* butterflies and the fruitfly *Drosophila melanogaster* as model organisms. Independent contrast analysis of *Vanessa* butterflies revealed significant correlations between eyespots 2 and 5 and between eyespots 3 and 4 on all wing surfaces (Table 1), suggesting the presence of an A-P colour pattern organizer centered on the M3 vein. *Drosophila* FLP/FRT wing clones do not cross a compartment boundary posterior to vein L5 (Fig. 2a,b), which is homologous to the M3 vein in butterflies (Fig. 1). A survey of lepidopteran mosaic gynandromorphs and homeotic mosaics with large clones suggests that a similar compartment boundary occurs in a homologous location (Fig. 2c). Collectively, these findings suggest the existence of a Far-Posterior compartment boundary and an associated positional organizer along the M3 vein between wing sectors 3 and 4 in holometabolous insect wings. We propose a model that provides a mechanistic explanation for both consistent phenotypic correlations between eyespots and the diverse evolutionary opportunities for individuation of serially homologous eyespots.

## Methods

### Independent contrast analysis of ocelli

Specimens were acquired from for 22 ingroup species from the genus *Vanessa* and 16 outgroup species from other genvera in tribe Nymphalini including *Aglais*, *Antanartia*, *Araschnia*, *Hypanartia*, *Kaniska*, *Mynes*, *Nymphalis*, *Polygonia*, and *Symbrenthia* ^5^. Outgroup taxa were selected with reference to Wahlberg *et al*.^46^. DNA was extracted from each specimen and 10 genes from the 38 exemplar species^46–48^ were PCR-amplified, Sanger-sequenced, and analyzed by Bayesian phylogenetic inference using MrBayes v3.2.1^49^ as described in detail in Abbasi and Marcus^5^.

Eyespot character states were primarily evaluated from specimens in the Marcus laboratory research collection as described previously^6^. For species represented by a small number of specimens, we supplemented our direct observations with examination of published photographs^50–55^. Species were examined to assess probable polymorphisms among regional populations, sexes, and seasonal forms.

Counting the number of parts within structures is a common way of measuring and comparing the non-hierarchical complexity of those structures^56–58^. Comparing the number of different colour components within each eyespot is a quantitative approach to determining the relative elaboration of eyespot phenotypes. Correlations between the number of colour components in eyespots on the same and different surfaces of the forewing and hindwing were evaluated by independent contrast (IC) analysis. IC analysis is a phylogenetic-based method that calculates differences in traits between two closely-related taxa by correcting for similarities caused by common ancestry^16,59^. The Bayesian phylogenetic tree was manipulated to remove all outgroups except *Hypanartia kefersteini*, which was used to root the tree as required by IC analysis. Branch lengths were estimated from the Baysian phylogenetic analysis and were found to meet the assumptions of the Brownian motion model. The data set was tested for phylogenetic signal using the Abouheif test^60^, found to exhibit phylogenetic autocorrelation, and so IC analysis was carried out using COMPARE 4.6b^61^ to remove autocorrelation using untransformed branch lengths. Statistical correlation was then used to analyze the contrasts and to determine relationships and correlation coefficients between each pair of traits.

### Clonal analysis of *Drosophila* wings

Two crosses were designed in order to create cellular clones (groups of cells with specific molecular or morphological characters distinguishable from surrounding cells) on the wing surfaces of *Drosophila melanogaster* labeled with phenotypic markers. Fly stocks were acquired from Bloomington Drosophila Stock Center at Indiana University (Bloomington, Indiana, USA). These fruit flies have a FLP (Flippase)/FRT (Flippase recognition target) mitotic recombination system integrated into different chromosomes in their genome^62^. The FLP/FRT system was used to produce mitotic clones (groups of cells homozygous for traceable phenotypic characters that stand in contrast to the background phenotype) in the wing discs of F1 females produced by controlled crosses (Extended Data Fig. 2). 72 to 96 hours after eggs were laid, a two 1-hour heat shocks (38 °C each), separated by a 1-hour room temperature interval (25 °C) were applied in order to induce the expression of the FLP enzyme. When FLP is expressed, the chromosomes undergo recombination at the FRT sites during the G2 phase of the cell cycle. When the cell divides, chromosomes randomly migrate into daughter cells and cells that receive two copies of the mutant yellow gene will express the mutant yellow phenotype. If a compartment boundary exists in vicinity of these cells, however, the marked cells would not be expected to cross the boundary.

Two separate FLP/FRT crosses were initiated: in the first cross, FRT sites and a copy of mutant yellow allele y^1^ were on chromosome 1 (Extended Data Fig. 3A). When heat shock was applied to the F1 progeny, the Flippase enzyme was expressed, resulting in recombination at the FRT sites in F1 females heterozygous for alleles at the *yellow* locus (*y* ^1^ */y* ^+^). FLP-induced recombination resulted in *y* ^+^ */y* ^+^ or *y* ^1^ */y* ^1^. The progeny of any cell that received two copies of the mutant yellow allele (*y* ^1^ */y* ^1^) showed the yellow cuticle phenotype of the homozygous mutant allele in a cell-autonomous fashion. In the second cross, the F1 females were homozygous for mutant yellow alleles on chromosome 1 (*y* ^1^ */y* ^1^) but also possessed one copy of the wild-type yellow allele *y* ^+^ and an FRT site on chromosome 2 (Extended Data Fig. 3B). This cross produced similar cellular clones marked by the absence of the wild-type yellow allele *y* ^+^ and the presence of the yellow phenotype by mitotic recombination on chromosome 2.

Flies were sorted by sex and screened for the presence of clones with an Olympus SZ61 stereomicroscope (Olympus, Tokyo, Japan) and a CO_2_ anesthetization system. Selected flies from the screening process were put into 1.6 mL microcentrifuge tubes containing 70% ethanol for slide preparation at a later time. Wings from each specimen were removed, fixed on slides with a drop of Euparal (Anglian Lepidopterist Supplies, Hindolveston, Norfolk, UK) and sealed with a cover slip. Dried slides were examined on a Leica M205 C stereomicroscope (Leica, Wetzlar, Germany) equipped with a Nikon Digital Sight DS-Fi2 imaging system (Nikon, Tokyo, Japan) under 160X magnification.

Slides with clones were photographed at 63X magnification. Wings that were grossly deformed and could not be mounted properly on a microscope slide were excluded from further analysis.

Mitotic clones on images of each wing were subsequently outlined (Fig. 2a) in Canvas 14 software (ACDSee, Fort Lauderdale, Florida, USA). Four landmarks at vein intersections (red dots) were used to perform a procrustes transformation^63^ of each wing so that the wings and their associated clones could be superimposed and compared (Fig. 2b).

### Mosaic gynadromorphs and homeotic mosaics in the Lepidoptera

The distribution of mitotic clones on the wings of Lepidoptera was examined in published reports of hundreds of naturally occurring and laboratory reared mosaic gynandromorphs and homeotic mosaics^22–24,64–81^. Most of these mosaic specimens have clones that were too small^74,75^, or inappropriately placed^73,79,80,82–84^ to be informative with respect to the wing compartment boundaries, but among those specimens with mitotic clones in the appropriate position, the F-P boundary was readily apparent in multiple specimens from the butterfly families Nymphalidae^23,24^ (Fig. 2c.), Papilionidae^72,83^, Pieridae^78^, and Lycaenidae^68^, and from the moth families Saturniidae^22,64,70^ and Lymantridae^76^. Similarly, the A-P boundary was apparent in specimens from the butterfly families Nymphalidae^24^, Papilionidae^72,83^, Pieridae^78^, Lycaenidae^68^, and Hesperiidae^80^, and from the moth families Saturniidae^22,64,70^ and Lymantridae^76^.

### Butterfly Culture and Immunohistochemistry

Adult *Junonia coenia* butterflies were collected from the Upper Green River Biological Preserve, Hart County, Kentucky, USA^85^ and allowed to oviposit and larvae were reared on *Plantago laceolata* host plants^86^. Wing imaginal discs were collected from 5^th^ instar *Junonia coenia* larvae at the j-hanging stage^87,88^ and immunohistochemistry experiments were performed using mouse 4F11 monoclonal anti-*En/Inv* ^89^ and anti-mouse Alexa 488-conjugated secondary (Molecular Probes, Eugene, Oregon, USA) antibodies according to established protocols^10^. The immuno-stained imaginal discs were then visualized with an Axioplan 2 epifluorescent microscope (Carl Zeiss, Oberkochen, Germany) equipped with a FITC filter and a 20X objective. Images were captured using a Zeiss AxioCam MRm camera (Fig. 3a).

### DNA sequence data

Sequences generated for phylogenetic analysis are deposited in Genbank (KJ648948-KJ649143 and KM225792-KM225794).

## Electronic supplementary material

Supplementary Information: Extended Data Figure 1–3
