# Supplementary material for: A new A-P compartment boundary and organizer in holometabolous insect wings
Source: Sci Rep. 2017 Nov 27;7:16337. doi: 10.1038/s41598-017-16553-5 (PMC5704014; doi:10.1038/s41598-017-16553-5)
Supplement: Supplementary file 1 — Supplementary Information: Extended Data Figure 1–3 [file 41598_2017_16553_MOESM1_ESM.doc]

**Supplementary Information**

for a

Research Article Resubmitted to Nature Scientific Reports

Submission Number: SREP-17-12523A

**A new A-P compartment boundary and organizer in holometabolous insect wings**

Roohollah Abbasi^1^ & Jeffrey M. Marcus^1^

^1^Department of Biological Sciences, University of Manitoba, Winnipeg, MB, Canada

**
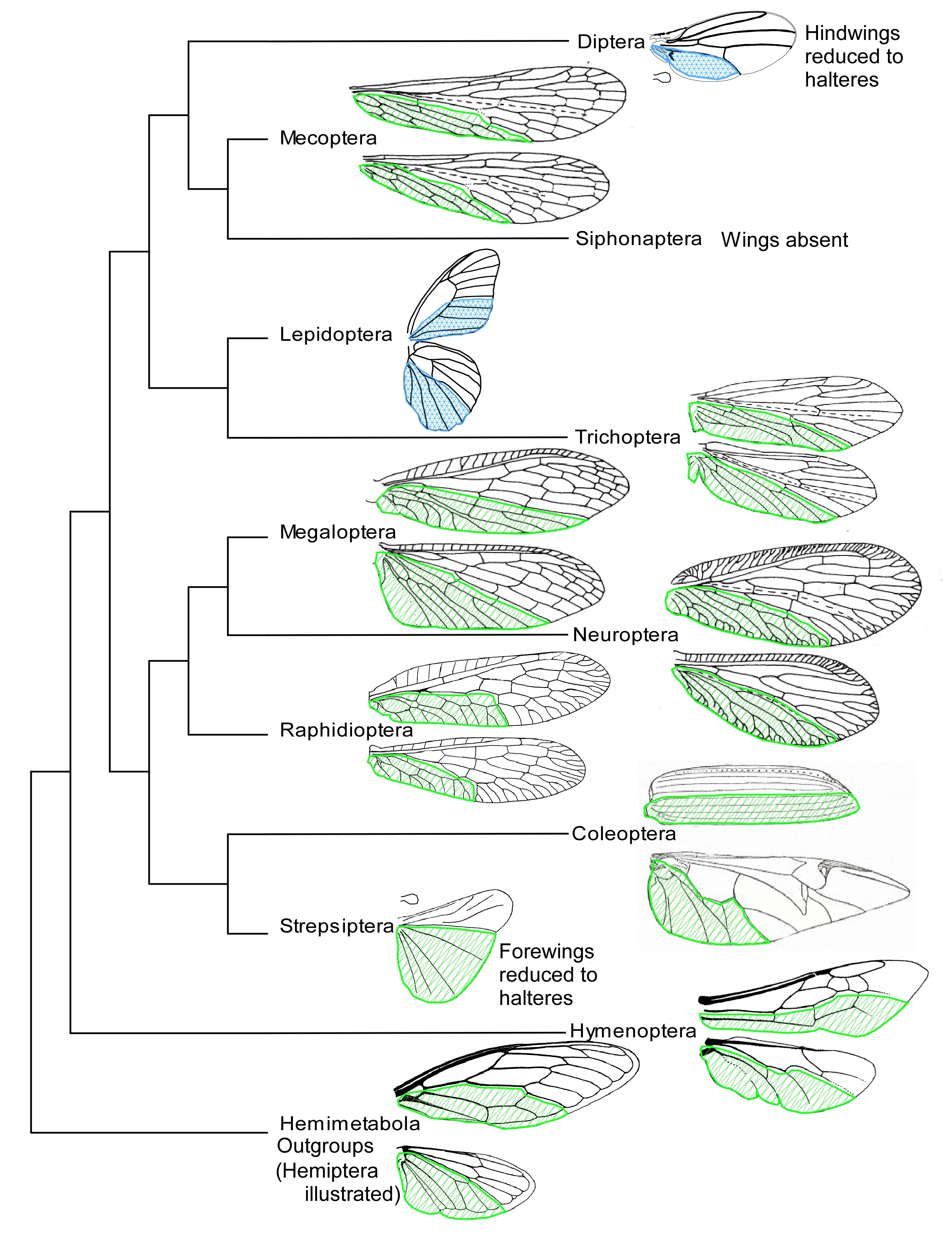
**

**Extended Data Figure 1 |** **Phylogeny of the holometabolous insect orders with wing phenotypes and inferred locations of the far posterior compartment** based on analysis of transcriptome sequences^1^ and depicted with arbitrary branch lengths. The wing venation patterns found in each insect order were redrawn from previous authors^2-5^. Blue crosshatching indicates the locations of far posterior wing compartments identified experimentally in this study. Green crosshatching indicates the predicted locations of the far posterior wing compartments in other insect orders if the position of the boundary is invariant relative to the position of homologous wing veins. The far posterior compartment is greatly reduced in the Diptera compared to the typical and ancestral state in holometabolous insects.

**
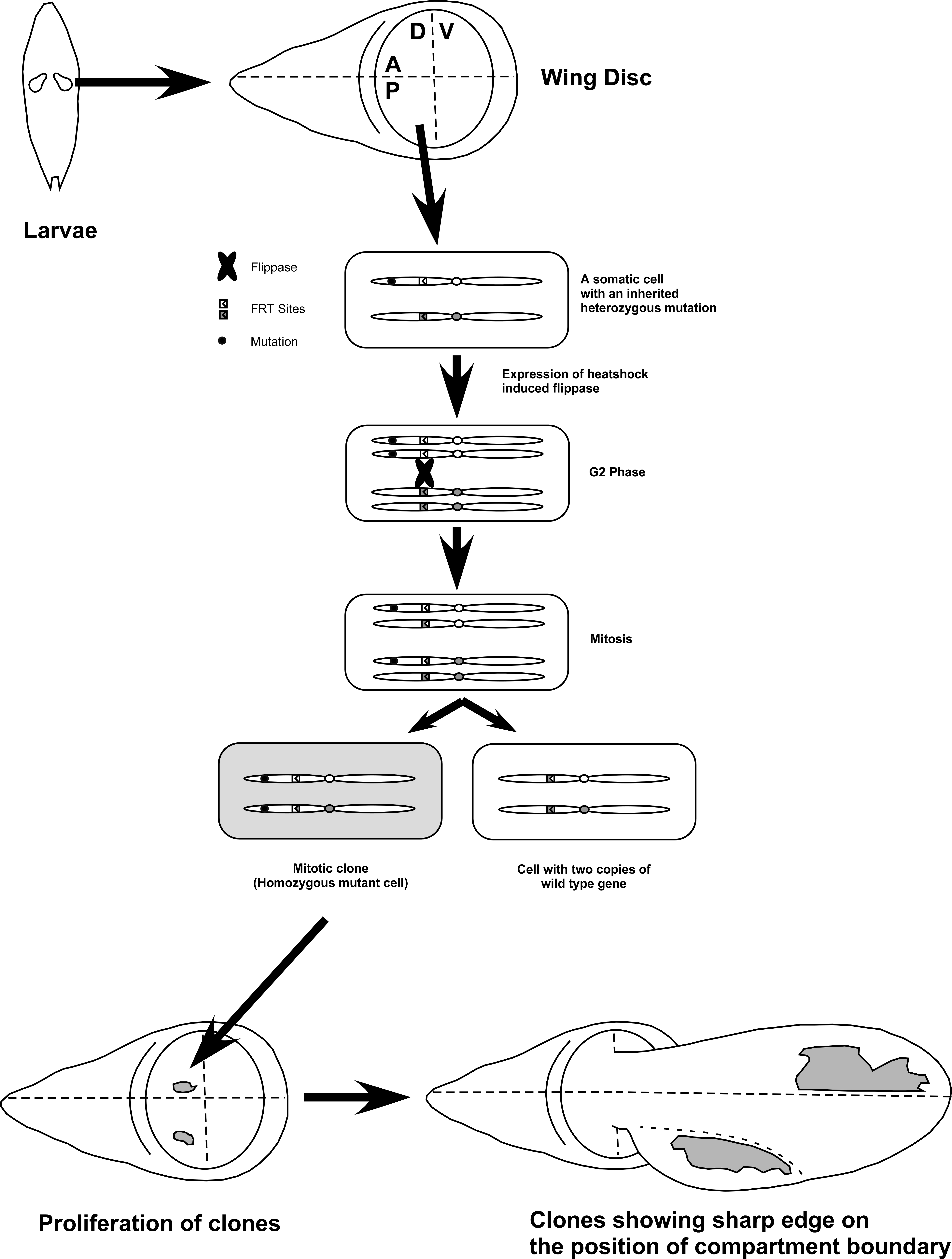
**

**Extended Data Figure 2 |** **Strategy for producing mitotic clones** using the FLP/FRT (Flippase/Flippase recognition target) system.

**
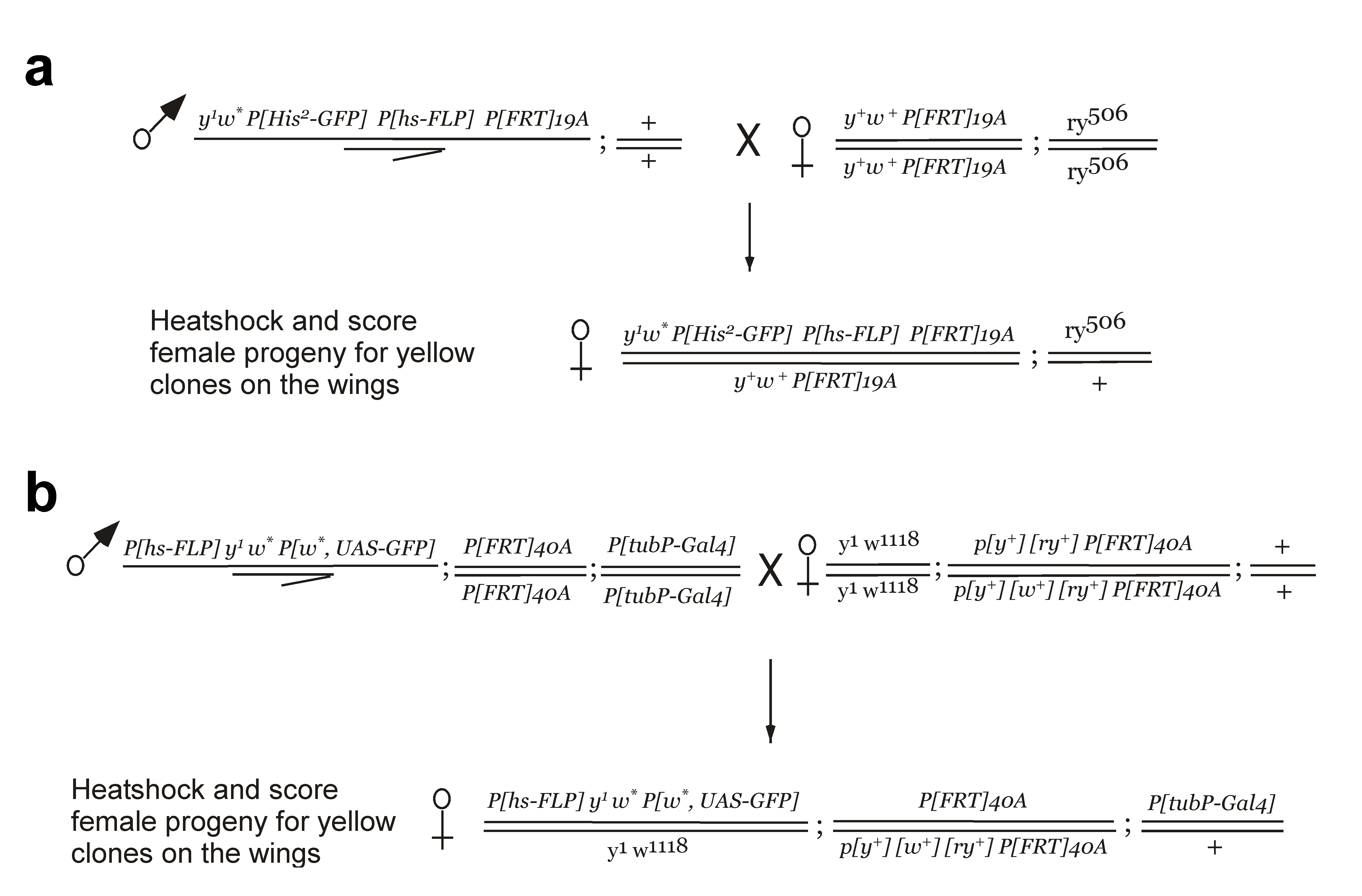
**

**Extended Data Figure 3 |** ***Drosophila* crosses** performed to create clones marked with *yellow* (*y^1^/y^1^*) to evaluate developmental compartment boundaries in the wing. **a,** Crosses to produce mitotic clones by means of mitotic recombination on chromosome 1. **b,** Crosses to produce mitotic clones by means of mitotic recombination on chromosome 2.

**References**

1 Peters, R. S. *et al.* The evolutionary history of holometabolous insects inferred from transcriptome-based phylogeny and comprehensive morphological data. *BMC Evol. Biol.* **14**, 52, doi:10.1186/1471-2148-14-52 (2014).

2 Wootton, R. J. Design, function and evolution in the wings of holometabolous insects. *Zoologica Scripta* **31**, 31-40, doi:10.1046/j.0300-3256.2001.00076.x (2002).

3 Comstock, J. H. *The Wings of Insects*. (Comstock Publishing Company, 1918).

4 Michener, C. D. Comments on minute Meliponini and the male of the genus *Pariotrigona* (Hymenoptera: Apidae). *J. Kansas Ent. Soc.* **74**, 231-236 (2001).

5 Rohdendorf, B. B. Evolution and classification of the flight apparatus of insects. *Trud. Paleontol. Inst. Akad. Nauk SSSR* **16**, 1-176 (1949).
